# Evaluating the Impact of Age-Related Macular Degeneration on Seasonal Affective Disorder: A Retrospective Cohort Study in a Chinese Population

**DOI:** 10.62641/aep.v53i5.1810

**Published:** 2025-10-05

**Authors:** Weijia Yang, Jun Jia, Xu Liu, Pengfei Wan

**Affiliations:** ^1^Department of Ophthalmology, The Second Affiliated Hospital of Xi’an Medical University, 710038 Xi’an, Shaanxi, China

**Keywords:** age-related macular degeneration, seasonal affective disorder, retrospective study

## Abstract

**Background:**

Age-related macular degeneration (AMD) usually affects the macular region of the retina, while seasonal affective disorder (SAD) is a complex mental disorder. However, the interaction between these two clinical conditions remains unexplored. Therefore, this study aimed to explore the influence of AMD on SAD and to assess the correlation of ocular pathology with lifestyle and mental health factors.

**Methods:**

This study recruited 158 AMD patients admitted to the Second Affiliated Hospital of Xi’an Medical University, China, between January 2020 and October 2023. Based on their affection status, the patients were divided into two groups: the SAD group (n = 58) and the non-SAD group (n = 100). Baseline characteristics, including blood pressure, hematological parameters, ocular parameters, and lifestyle factors, were compared between the two groups to evaluate the potential influence of AMD on SAD.

**Results:**

We observed specific differences in the family history of mental illness between the non-SAD and SAD groups (*p* < 0.001). However, the two groups’ other baseline characteristics, such as blood pressure and hematological parameters, were comparable (*p* > 0.05). Additionally, significant differences were also observed in central retinal thickness (CRT), choroidal thickness, lesion atrophy area, and macular volume between the two groups (*p* < 0.001). Moreover, intraocular pressure (IOP) did not reveal a significant difference between the two groups (*p* > 0.05). Compared with the SAD group, the non-SAD group had significantly better vision, longer exercise duration, sunlight exposure time, outdoor activity, and lower sedentary behavior (all *p* < 0.001). The logistic regression analysis indicated that increased macular volume (odds ratio (OR) = 3.054, *p* = 0.008) and sedentary behavior (OR = 4.382, *p* < 0.001) significantly increased SAD risk. Additionally, the absence of a family history of mental illness did not reach statistical significance (OR = 0.375, *p* = 0.129), but a specific correlation was still observed.

**Conclusion:**

This study shows a correlation between SAD and AMD. The significant differences in ocular pathological characteristics, lifestyle factors, and mental health status between the SAD and non-SAD groups suggest the crucial role of visual function and lifestyle in regulating mood and circadian rhythm in AMD patients.

## Introduction

Age-related macular degeneration (AMD) is a progressive and debilitating eye disease that poses a substantial public health burden, especially among the elderly [1, 2]. AMD, a leading cause of visual impairment and blindness, results in the loss and injuries of central vision due to irreversible damage to the macula [3, 4], affecting millions worldwide in the elderly [5, 6]. Besides vision loss, AMD is linked to anxiety and depression. A systematic review shows that approximately 15.7% to 44% of AMD patients experience depression symptoms, and 9.6% to 30.1% suffer from anxiety [7]. These mental health issues are mainly associated with the visual impairment caused by AMD, which adversely affects the overall functional abilities of the patients [8, 9]. As the anxiety levels in AMD patients increase, their quality of life significantly declines, potentially impacting their mental health [10] and even leading to more psychological conditions.

Furthermore, seasonal affective disorder (SAD), a subtype of depression, is characterized by the onset of major depressive episodes in specific seasons, usually autumn and winter [11, 12]. Usually, SAD results from a complex interaction among genetic, environmental, and physiological factors [13]. Studies have reported that reduced sunshine exposure, disrupted circadian rhythm, and altered neurotransmitter levels, especially serotonin, are strongly associated with its pathophysiology [14, 15]. SAD causes significant psychological distress in patients and results in adverse impacts on physical function and overall quality of life. Clinical studies have shown that patients with impaired vision experience significantly higher incidence rates of depression, anxiety, and sleep disorders compared to the general population [16, 17, 18].

However, limited studies have investigated SAD incidence in patients with specific ophthalmic diseases, particularly regarding any potential association between AMD and SAD. Therefore, this retrospective study explored the potential impact of AMD on SAD development within a Chinese population, aiming to provide personalized treatment options and improve patient outcomes in clinical practices.

## Materials and Methods

### Study Participants

This study recruited 158 AMD patients admitted to the Second Affiliated Hospital of Xi’an Medical University, China, between January 2020 and October 2023. Based on their affection status, the patients were divided into the SAD group (n = 58) and the non-SAD group (n = 100).

### Inclusion and Exclusion Criteria

Inclusion criteria were as follows: (i) individuals aged 50–80 years with confirmed AMD diagnosis through slit-lamp examination, fundus photography, optical coherence tomography (OCT), and fundus fluorescein angiography (FFA), (ii) patients with baseline central macular thickness (CMT) of ≥300 µm as assessed by OCT, (iii) those with normal psychological and cognitive function, and (iv) patients with complete medical records.

However, exclusion criteria included patients with ocular tumors, a history of eyeball enucleation, diseases affecting the extramacular retina or choroidal atrophy, blindness or death before the end of the follow-up period, other eye diseases (cataract, diabetic retinopathy, and macular epiretinal membrane), a history of macular surgery, laser or radiation therapy, severe cardiovascular, hepatic, or renal diseases, other malignant tumors, and those with no OCT examination.

### Diagnostic Criteria of SAD 

SAD is diagnosed as follows:

(1) A consistent temporal relationship is observed between the onset of major depression in major depressive disorder and a specific time of the year (e.g., autumn or winter). (2) Complete remission or change from major depression to mania or hypomania, also occurs at a specific time of year (e.g., depression subsides in spring). (3) A seasonal relationship can be observed if two episodes of major depression occur in the past two years, with no non-seasonal major depression during this period. (4) The onset of seasonal major depression, as described, is significantly more frequent than non-seasonal major episodes over an individual’s lifetime [19].

### Patients Observation and Data Collection

#### Baseline Characteristics

Baseline characteristics such as age, sex, body mass index (BMI), family history of AMD, family history of mental illness, smoking history, excessive drinking history, cardiovascular disease history, marital status, education status, monthly income level, and place of residence were collected from the medical record system of the Second Affiliated Hospital of Xi’an Medical University, China.

#### Blood Pressure and Hematological Parameters 

The systolic pressure (SP; normal pressure: 90–140 mmHg), diastolic pressure (DP; normal value: 60–90 mmHg), white blood cell (WBC; normal value: 4.0–10.0 × 10^9^/L), hemoglobin (HB; normal value: 120–160 g/L in male, and 110–150 g/L in female), platelets (PLTs; normal value: 100–300 × 10^9^/L), and C-reactive protein (CRP; normal value: <8 mg/L) levels of each patient were accessed from medical record system during hospitalization.

#### Ocular Parameters 

The ocular parameters for both groups of patients were obtained from the medical record system, including vision, intraocular pressure (IOP), central retinal thickness (CRT), choroidal thickness, lesion atrophy area, and macular volume. During hospitalization, the uncorrected vision was evaluated using the LogMAR vision chart, where a higher value represented poorer vision; contrast sensitivity (CS) was determined using a visual function analyzer (Optec 6500; Stereo; Chicago, IL, USA) under natural daylight conditions without glare at a 6-meter distance. CS values of low, middle, and high spatial frequencies in both eyes were measured, with each spatial frequency measured 2–3 times to obtain the average.

The analysis focused on measurements from poorer eyes. IOP was accurately measured using a tonometer, and findings were compared to the reference range of 11–21 mmHg. OCT was performed using a rapid macular scanning program, focusing on a 6 mm × 6 mm around the central fovea and divided into concentric circles with 1-, 3-, and 6-mm diameters. Using special software, CRT was determined as the vertical distance between the retinal pigment epithelium (RPE) and the inner boundary, and the macular volume was calculated accordingly. Choroidal thickness under the central fovea was measured in the horizontal and vertical directions, with each point measured three times to obtain an average.

Two trained ophthalmologists independently conducted multimodal imaging using specialized software. Image J software (version 1.49b; National Institutes of Health; Bethesda, MD, USA) was used to calibrate different imaging modalities. Employing the Heidelberg image, a standard scale of 200 µm was set, the lesion contour was delineated, and the atrophy area was calculated based on the standardized scale.

#### Lifestyle 

Information about weekly exercise hours, sunlight exposure hours, outdoor time, and sedentary hours for both patient groups was collected from the hospital’s medical record system. The data collection process was as follows: upon arrival at the hospital, a lifestyle questionnaire was provided to each patient. A professional researcher was available to explain and help them in filling the data. The patients were asked to return the questionnaire by their next visit after seven days, and the data were then recorded into the system. As detailed in Table 1, the lifestyle questionnaire included four items. Weekly exercise hours were determined by summing the daily exercise hours, while daily average hours for sunlight exposure, outdoor activity, and sedentary time were obtained by dividing the total hours by the number of days.

**Table 1.** **Items of the lifestyle questionnaire**.

Item	Day 1	Day 2	Day 3	Day 4	Day 5	Day 6	Day 7
1. How many hours a day do you exercise?							
2. How much sunlight do you receive each day?							
3. How many hours a day do you exercise outdoors?							
4. How many hours a day are you sedentary?							

### Statistical Analysis 

Statistical analysis was performed using SPSS 26.0 software (IBM; Armonk, NY, USA). Categorical variables were expressed as [n (%)]. The chi-square test was applied with the basic formula when the sample size was ≥40 and the theoretical frequency T was ≥5, using χ^2^ as the test statistic. An adjusted chi-square test with a correction formula was applied for sample size ≥40 and the theoretical frequency of 1 ≤ T < 5. The Fisher’s exact test was applied if the sample size was <40 or T <1.

However, continuous variables were checked for normal distribution using the Shapiro-Wilk method. Normally distributed data were expressed as mean ± SD (standard deviation) and analyzed using a *t*-test, while non-normal distribution was presented as median (M) (P_25_, P_75_) and analyzed using the Mann-Whitney U test.

Furthermore, Pearson correlation coefficient analysis was conducted to select independent variables, with the correlation coefficient (r) ranging from –1 to 1, with |r| > 0.4 indicating a high correlation. Variables with high correlation to many other variables were considered unsuitable for regression analysis due to multicollinearity. A tolerance value below 0.1 suggests severe multicollinearity, as tolerance measures the number of variances not explained by other variables. The variance inflation factor (VIF) indicates inflation due to multicollinearity. Generally, VIF above 10 indicates severe multicollinearity, while a VIF between 5 and 10 suggests moderate multicollinearity. Binary logistic regression was used to analyze the potential influence of AMD on SAD, with a bilateral *p*-value of <0.05 indicating statistical significance.

## Results

### Comparison of Baseline Characteristics Between the Two Groups

The baseline characteristics of the study participants are shown in Table 2. We observed that these characteristics, including age, sex, BMI, smoking history, excessive drinking history, cardiovascular disease history, marital status, education status, monthly income level, and place of residence, were comparable between the non-SAD and SAD groups (*p* > 0.05). However, there was a significant difference in the family history of mental illness between the two groups (*p* < 0.001).

**Table 2.** **Comparison of baseline characteristics between the two groups [(mean ± SD)/n (%)]**.

Parameters	Non-SAD group (n = 100)	SAD group (n = 58)	*t*/χ^2^	*p*-value
Age (years)	66.40 ± 6.37	67.79 ± 7.34	1.253	0.212
Sex (male/female)	47 (47.00)/53 (53.00)	31 (53.45)/27 (46.55)	0.611	0.435
BMI (kg/m^2^)	24.36 ± 1.94	24.13 ± 1.94	0.717	0.475
Smoking history (No/Yes)	82 (82.00)/18 (18.00)	48 (82.76)/10 (17.24)	0.014	0.904
Excessive drinking history (No/Yes)	73 (73.00)/27 (27.00)	43 (74.14)/15 (25.86)	0.024	0.876
Cardiovascular disease history (No/Yes)	77 (77.00)/23 (23.00)	41 (70.69)/17 (29.31)	0.773	0.379
Marital status (married/single)	79 (79.00)/21 (21.00)	41 (70.69)/17 (29.31)	1.388	0.239
Education status (years)	10.09 ± 3.12	10.43 ± 4.07	0.551	0.583
Monthly income level (CNY)	5782.05 ± 1482.38	5826.04 ± 1219.58	0.191	0.848
Place of residence (village/city)	47 (47.00)/53 (53.00)	20 (34.48)/38 (65.52)	2.355	0.125
Family history of mental illness (No/Yes)	92 (92.00)/8 (8.00)	38 (65.52)/20 (34.48)	17.657	<0.001

Note: Family history of mental illness refers to the presence of mental illnesses like schizophrenia, depression, anxiety, obsessive-compulsive disorder, mania, mental retardation, Alzheimer’s disease, and sexual and psychological disorders among family members across three generations on the paternal, maternal, direct, or collateral line. Smoking history refers to continuous or cumulative smoking lasting over six months. Excessive drinking history refers to long-term alcohol consumption over 5 years (equivalent to ≥40 g/d ethanol for males and ≥20 g/d for females) or excessive alcohol intake within 2 weeks (equivalent to >80 g/d ethanol). SD, standard deviation; SAD, seasonal affective disorder; BMI, body mass index. The exchange rate is 1 USD = 6.48 CNY.

### Comparison of Blood Pressure and Hematological Parameters 

The SP, DP, HB, PLT, WBC, and CRP levels were comparable between the two groups (*p* > 0.05), as shown in Table 3.

**Table 3.** **Comparison of blood pressure and hematological parameters between the two groups (mean ± SD)**.

Parameters	Non-SAD group (n = 100)	SAD group (n = 58)	*t*	*p*-value
SP (mmHg)	128.73 ± 8.85	131.02 ± 11.86	1.277	0.205
DP (mmHg)	79.71 ± 6.89	80.95 ± 7.42	1.058	0.292
HB (g/L)	134.76 ± 10.75	136.86 ± 9.41	1.239	0.217
PLT (×10^9^/L)	262.91 ± 21.99	267.07 ± 19.23	1.198	0.233
WBC (×10^9^/L)	6.75 ± 0.53	6.69 ± 0.38	0.692	0.490
CRP (mg/L)	3.92 ± 1.11	4.18 ± 1.20	1.366	0.174

Note: SP, systolic pressure; DP, diastolic pressure; WBC, white blood cell; HB, hemoglobin; PLT, platelet; CRP, C-reactive protein.

### Comparison of Ocular Parameters 

Table 4 describes the ocular parameters of the two groups. The non-SAD group showed a significantly better vision than the SAD group (*p* < 0.001). Furthermore, significant differences were observed in CRT, choroidal thickness, lesion atrophy area, and macular volume between the two groups (*p* < 0.001), with no significant difference in IOP (*p* > 0.05). These findings suggest a potential association between SAD and changes in ocular parameters, highlighting the potential impact of AMD on SAD.

**Table 4.** **Comparison of ocular parameters between the two groups [M (P_25_, P_75_)]**.

Parameters	Non-SAD group (n = 100)	SAD group (n = 58)	Z	*p*-value
LogMAR value	0.30 (0.20, 0.30)	0.30 (0.30, 0.40)	–7.331	<0.001
IOP (mmHg)	14.54 (13.13, 16.39)	14.07 (13.14, 15.01)	–1.605	0.108
CRT (µm)	253.92 (245.40, 262.06)	336.99 (324.79, 344.36)	–10.461	<0.001
Choroidal thickness (µm)	173.78 (168.04, 184.18)	210.91 (198.03, 224.92)	–10.075	<0.001
Lesion atrophy area (mm^2^)	0.49 (0.44, 0.54)	0.71 (0.64, 0.81)	–9.987	<0.001
Macular volume (mm^3^)	6.15 (5.61, 6.68)	6.57 (6.28, 6.83)	–4.078	<0.001

Note: IOP, intraocular pressure; CRT, central retinal thickness.

### Comparison of Lifestyle Between the Two Groups

The non-SAD group showed significantly longer exercise duration, sunlight exposure, outdoor activities, and lower sedentary behavior compared with the SAD group (all *p* < 0.001, Table 5).

**Table 5.** **Comparison of lifestyle between the two groups [M (P_25_, P_75_)]**.

Parameters	Non-SAD group (n = 100)	SAD group (n = 58)	Z	*p*
Exercise duration (h/w)	3.30 (2.90, 3.70)	2.65 (2.30, 2.90)	–8.008	<0.001
Sunshine irradiation duration (h/d)	1.40 (1.20, 1.80)	0.90 (0.90, 1.00)	–10.352	<0.001
Outdoor time (h/d)	2.60 (2.30, 2.70)	1.85 (1.50, 2.20)	–9.045	<0.001
Sedentary behavior (h/d)	5.40 (4.55, 6.20)	6.60 (5.80, 7.30)	–6.792	<0.001

### Selection of Predictive Factors

The collinearity diagnostics indicated that all variables had VIF values below 10, indicating no severe multicollinearity. However, CRT showed a higher VIF (7.007), suggesting moderate collinearity that requires attention (Table 6). A correlation analysis was performed on the statistically significant indicators in the univariate analysis. Results revealed that LogMAR value, CRT, Choroidal thickness, Lesion atrophy area, sunlight exposure duration, exercise duration, and outdoor time were highly correlated, making them unsuitable for inclusion in the regression analysis (Table 7).

**Table 6.** **Collinearity diagnostics results**.

Parameters	Tolerance	VIF
Family history of mental illness	0.942	1.061
LogMAR value	0.647	1.546
CRT	0.143	7.007
Choroidal thickness	0.342	2.920
Lesion atrophy area	0.382	2.617
Macular volume	0.863	1.158
Exercise duration	0.564	1.774
Sunshine irradiation duration	0.476	2.102
Outdoor time	0.473	2.114
Sedentary behavior	0.652	1.534

Note: Dependent Variable: The presence or absence of SAD is central retinal thickness (CRT), and variance inflation factor (VIF).

**Table 7.** **Correlation analysis**.

Parameters	Family history of mental illness	LogMAR value	CRT	Choroidal thickness	Lesion atrophy area	Macular volume	Exercise duration	Sunshine irradiation duration	Outdoor time	Sedentary behavior
Family history of mental illness	1.000	–0.019	0.146	0.102	0.020	0.069	–0.181*	–0.098	–0.116	0.045
LogMAR value	–0.019	1.000	–0.563**	–0.458**	–0.435**	–0.072	0.398**	0.429**	0.357**	–0.312**
CRT	0.146	–0.563**	1.000	0.803**	0.775**	0.346**	–0.635**	–0.703**	–0.682**	0.573**
Choroidal thickness	0.102	–0.458**	0.803**	1.000	0.624**	0.232**	–0.462**	–0.610**	–0.573**	0.452**
Lesion atrophy area	0.020	–0.435**	0.775**	0.624**	1.000	0.262**	–0.510**	–0.592**	–0.599**	0.433**
Macular volume	0.069	–0.072	0.346**	0.232**	0.262**	1.000	–0.121	–0.157*	–0.159*	0.293**
Exercise duration	–0.181*	0.398**	–0.635**	–0.462**	–0.510**	–0.121	1.000	0.569**	0.470**	–0.365**
Sunshine irradiation duration	–0.098	0.429**	–0.703**	–0.610**	–0.592**	–0.157*	0.569**	1.000	0.563**	–0.354**
Outdoor time	–0.116	0.357**	–0.682**	–0.573**	–0.599**	–0.159*	0.470**	0.563**	1.000	–0.382**
Sedentary behavior	0.045	–0.312**	0.573**	0.452**	0.433**	0.293**	–0.365**	–0354**	–0.382**	1.000

Note: CRT, central retinal thickness. ** means significant correlations at *p* < 0.01 level; * means significant correlations at *p* < 0.05 level.

### Logistic Regression Analysis of SAD and Various Parameters 

The logistic regression analysis showed that increased macular volume (OR = 3.054, *p* = 0.008) and increased sedentary behavior (OR = 4.382, *p* < 0.001) were significantly associated with a higher risk of SAD. However, the absence of a family history of mental illness did not reach statistical significance (OR = 0.375, *p* = 0.129) (Table 8).

**Table 8.** **Logistic regression analysis of the potential influence on SAD**.

Parameters	B	Standard error	Wald	*p*-value	OR	95% CI	
						Lower limit	Upper limit
Family history of mental illness (reference: Yes)	–0.981	0.647	2.300	0.129	0.375	0.105	1.332
Macular volume	1.116	0.421	7.028	0.008	3.054	1.338	6.970
Sedentary behavior	1.478	0.273	29.216	<0.001	4.382	2.565	7.489

CI, confidence interval; OR, odds ratio.

## Discussion

This study explored the potential impact of AMD on SAD, indicating that increased macular volume and sedentary behavior significantly elevated the risk of SAD. Specifically, AMD patients facing declining vision and impaired visual function may experience a reduction in quality of life, potentially inducing or exacerbating SAD. Moreover, sedentary behavior emerged as a critical risk factor, closely associated with reduced physical activity and insufficient sunlight exposure, known as SAD triggers. Although the association between a family history of mental illness and SAD did not reach statistical significance, a certain correlation was still observed, suggesting a potential genetic component in SAD. Overall, these results provide novel insights into the mental health challenges faced by AMD patients, emphasizing the potential impact of visual health and lifestyle on SAD risk.

The analysis of ocular parameters between the non-SAD and SAD groups showed significant differences. Compared to the SAD group, the non-SAD group exhibited better vision, fewer retinal and choroidal thickness changes, and a smaller lesion atrophy area. This finding aligns with the pathophysiology of AMD, which manifests as progressive macular degeneration and consequent visual impairment [20, 21]. Significantly, decreased visual function in AMD patients is associated with increased depression risk and reduced quality of life [22, 23]. Impaired vision may limit outdoor activities and exposure to natural light, factors known to affect mood and SAD development [24, 25]. Retinal photoreceptors regulate vision, circadian rhythms, and emotion balance [26]. The retinal and choroidal thickness changes observed in AMD patients may disrupt the photoreceptor function, inducing circadian rhythm disruptions and neurotransmitter system disorders that impact emotional regulation. This mechanism may lead to depressive symptoms, especially SAD. Reducing natural light exposure further aggravates circadian rhythm disruption, affecting emotional health [27]. Visual impairment can also reduce functional independence, social interactions, and overall quality of life, leading to loneliness and psychological distress, which increases SAD risk in AMD patients [28]. Therefore, we speculate that changes in eye parameters may be associated with SAD development in AMD patients.

Furthermore, physical activity, sunlight exposure, and outdoor time are closely related to SAD development. Sunlight irradiation plays a crucial role in regulating circadian rhythms, serotonin levels, and vitamin D synthesis, which impact mood regulation and SAD risk [29]. In this study, significant differences were found in lifestyle between the two groups, with the non-SAD group engaging in more physical activities, sunlight irradiation, and outdoor time than the SAD group, suggesting that an active lifestyle may have helped prevent SAD. On the contrary, increased sedentary behavior is a negative factor linked to a high incidence of SAD, potentially aggravating AMD progression and creating a vicious circle. These lifestyle differences between the two groups highlight the potential role of lifestyle changes in managing AMD and preventing SAD in this population. Although significant differences were found in the family history of mental illness, LogMAR value, CRT, choroidal thickness, lesion atrophy area, macular volume, exercise duration, sunlight exposure, outdoor time, and sedentary behavior, logistic regression analysis revealed that none of these variables were independent risk factors for SAD in AMD patients. The specific pathogenesis remains unclear and needs further research.

From a clinical perspective, these observations provide a new idea for managing AMD patients. Beyond routine ophthalmic treatment, physicians should monitor the mental health status of patients, especially those with a family history of mental illness. Meanwhile, encouraging patients to engage in more physical activities, prolong outdoor activity time, and reduce sedentary behavior might reduce SAD risk and improve the overall quality of life.

Besides some valuable insights, this study had certain limitations. Firstly, as a retrospective, single-center study, it may have selection bias and limited generalizability. Secondly, recall bias or inaccuracies may affect the self-reported lifestyle data. Future multi-center prospective studies are needed to expand these findings. Moreover, this study opens future research avenues, such as exploring shared genetic variations between AMD and SAD and their influence on the pathogenesis and clinical manifestations. Furthermore, assessing lifestyle factors’ independent and combined effects on AMD and SAD could help determine the most effective interventions.

## Conclusion

In summary, a complex association exists between AMD and SAD. Patients with SAD after AMD show significant differences compared to non-SAD patients, especially in family history of mental illness, decreased visual function, changes in retinal and choroidal structure, exercise duration, sunlight exposure, and outdoor time. These observations highlight the crucial role of visual function in mood regulation and circadian rhythm control and the potential preventive impact of an active lifestyle against SAD. Further multi-center studies are required to validate and investigate these associations.

## Availability of Data and Materials

The datasets used and/or analyzed during the current study were available from the corresponding author on reasonable request.
